# Health-related factors correlate with behavior trends in physical activity level in old age: longitudinal results from a population in São Paulo, Brazil

**DOI:** 10.1186/1471-2458-10-690

**Published:** 2010-11-10

**Authors:** Marcela T Ferreira, Sandra MM Matsudo, Manoel CSA Ribeiro, Luiz R Ramos

**Affiliations:** 1 Department of Internal Medicine and Therapeutic, São Paulo Federal University, São Paulo, Brazil; 2 Physical Fitness Research Laboratory from São Caetano do Sul, São Caetano do Sul, Brazil; 3 Departament of Social Medicine, Santa Casa da Misericórdia, São Paulo, Brazil; 4 Department of Preventive Medicine, São Paulo Federal University, São Paulo, Brazil

## Abstract

**Background:**

Physical inactivity in leisure time is common among elderly in Brazil and this finding is particularly alarming considering that this population is greatly affected by chronic diseases. The identification of health factors that influence physical activity level (PAL) will help in the development of strategies for increasing PAL older adults. The current research aimed to identify variables that independently affect behavior trends in PAL over the course of two years among elderly.

**Methods:**

A survey entitled the Epidoso Project ("Epidemiology of aging") studied 1,667 community-based older individuals in São Paulo city, Brazil over the course of two years. Physical activity level was determined through questions about frequency and duration of physical activities. Body Mass Index was calculated; functional capacity was assessed through the ADL (activities of daily living) scale; cognition was assessed by Mini-Mental State Examination; and mental health was assessed through the Dysthymia Screening. Experiences of falls and fractures were also assessed. Subjects were divided into three groups according to their self-report of Physical Activity Level: a - Regularly Active; b - Insufficiently Active and c - Physically Inactive. Behavior trends in PAL were also measured after two years. Multivariate regression model methodology was used to test associations longitudinally.

**Results:**

Results from the final model demonstrated that the risk of a not favorable behavior trend in PAL, which included the group who remained physically inactive and the group that displayed decreased PAL, in this cohort of older adults was significantly increased if the individual was female (OR = 2.50; 95% CI = 1.60-3.89; *P < 0.01*), older (80 y vs. 65 y, OR = 6.29, 95% CI = 2.69-14.67; *P < 0.01*), dependent on help from others for activities in the ADL scale (moderate-severe = 4-7+ vs. 0 ADLs) (OR = 2.25, 95% CI = 1.20-4.21; *P < 0.011*) or had experienced a history of falls with consequences (OR = 6.88, 95% CI = 0.91-52.01; *P < 0.062*).

**Conclusions:**

Age, gender, ADL scores and falls were associated with a not favorable behavior trend in PAL. Promotion programs should target these factors, reducing barriers to achieve desired changes in PAL.

## Background

An increase in the proportion of elderly in the population has been reported in developed [1,2] and developing countries [3-6]. The number of people over 60 years old is projected to double in the next 20 years. According to demographic projections, 33 million Brazilians will be older than 60 years in 2025 [1]. Furthermore, the prevalence of physical inactivity is increasing worldwide as a result of changes in individuals' lifestyles. Studies conducted in Brazil reported that nearly 45.0% of the people [7,8] and 50.1% of the elderly are sedentary [8]. Data from a national survey called VIGITEL (" "Surveillance of Risk and Protective Factors for Chronic Diseases by Telephone Interviews") in all capitals of the 26 Brazilian states found that only 12.7% of the elderly were involved in physical activity in leisure time, whereas 56.5% were classified as physically inactive [9]. A recent report from the World Health Organization (WHO) demonstrates that physical inactivity is the fourth-leading global risk factor for mortality [10] and is one of the most important modifiable risk factors for noncommunicable diseases (NCDs), such as heart disease, diabetes and cancer, contributing substantially to the global burden of disease, disability and death. Physical activity can prevent and help treat NCDs and maintain physical and mental health and quality of life in elderly adults [10-12]. Moreover, low levels of physical activity have been associated with high health care costs [13]. Therefore, national data on the prevalence of and factors related to physical activity level may aid in identifying effective public health measures, consequently lowering health care charges.

Population studies have been conducted in developed and developing countries on physical activity and health-related factors among the elderly [14-18]. However, few published studies have specifically investigated physical activity level determinants, among older adults in Brazil [7,8,19,20].

The Epidoso ("Epidemiologia do envelhecimento" - "Epidemiology of aging") Project was the first longitudinal study of the elderly in São Paulo, a large urban center in Brazil. This study has followed elderly people since 1990, searching for factors associated with healthy aging and risk factors for mortality. This project has resulted in important contributions, through some papers in the area of risk factors for mortality, functional capacity, falls and noncommunicable diseases, as well as the prevalence of several diseases in this population [4,5,21]. Therefore, we used data from the Epidoso Project to identify health-related factors that independently influence behavior trends in physical activity level over the course of two years among the elderly. As far as we know, no study has been conducted with the Brazilian elderly that underlines factors that influence trends in PAL over two years.

## Methods

### Design and subjects

Epidoso Project was a longitudinal study of older adults living in *Saúde district *in São Paulo city (Brazil) that has aimed to identify predictors of mortality among the elderly since 1991. A previous census had established that the *Saúde *district included a population from different socioeconomic backgrounds in a residential area with a low rate of immigration. All the elderly aged 65 and over living in the area of the Center for the Study of Aging (CSA) at the Paulista Medicine School of the Federal University of São Paulo (EPM/UNIFESP), which is located in this district area, were enrolled in the study. A total of 1,667 people met the age criterion and were successfully interviewed (mean age: 74.9 ± 6.7 years). Subjects have been tracked at home (household survey) in 1991/1992 (baseline) and were invited to attend a clinical examination in CSA. After the first stage, sample was followed up on an outpatient basis with routine assessments every 6 months during the study period, with access to a multidisciplinary team. The same methodology was repeated after two years in 1994/1995 (follow-up) and no intervention procedure was conducted during this period [21]. The average length of follow-up was 24 months (minimum of 18 and maximum of 38). The selection method and inclusion criteria for the sample can be found in previously published papers [4,21-23].

Of the initial sample, only 860 (51.2%) were included in the analysis (respondents).The 48.8% of participants (n = 821) who did not provide baseline measures or refused to participate were not similar in gender and age but were similar in education and physical activity level compared with the respondents (n = 865). Those with missing data were 61.6% (n = 497) female, 51.5% (n = 415) had completed primary school, 50.4% (n = 367) were aged between 70 and 79 years old and 87.4% (n = 513) were classified as physically inactive. The maximum number of missing values from initial sample was 1,235 for BMI, while all other variables had fewer than 902 values missing.

The household surveys followed a structured questionnaire - BOMFAQ (Brazilian OARS Multidimensional Functional Assessment Questionnaire, which was conceived in the United States (Duke University Center for the Study of Aging and Human Development, 1978), published by Ramos & Goihman [23] and formerly used in cross-sectional studies [24,25]. This questionnaire provides socio-demographic data (i.e., age, gender, marital status, education level, and per capita income in the household in US$/month) and assesses the individuals' subjective perception of physical and mental health (cognitive and emotional aspects), dependence on help from others for activities of daily living, daily practice of physical activities during leisure time, and social and family support. Participants were interviewed by trained professionals using this instrument.

The Ethics Committee of the Federal University of São Paulo approved the study protocol, and written informed consent was obtained from all individuals (CEP 1537/06).

### Measures

#### Physical activity level assessment

Physical activity level (PAL) was determined by self-report of the involvement (or lack thereof) in regular physical activities during leisure time. In case of a positive answer, subjects were requested to indicate frequency (times per week) and average duration (minutes per session). Data from the questionnaire provided values for frequency and duration of physical activities but not the intensity. It was assumed that PA reported in the questionnaire could be classified as moderate, based on several studies demonstrating that most of the elderly perform activities that are light to moderate in intensity [7,26,27]. Subsequently, the sample was classified into three groups according to the recommended PA level from American College of Sports Medicine [12] and Center of Disease and Prevention Control (CDC) [28] as detailed below.

Classification of physical activity level

a - Regularly Active: those who reported at least 5 days per week and at least 30 minutes per day of any type of PA;

b - Insufficiently Active: subjects who reported less than 5 days per week and <30 minutes per day and;

c - Physically Inactive: subjects that reported no participation in any moderate-to-vigorous physical activity in leisure time.

Then, changes or maintenance in physical activity level over the course of two years were defined as "behavior trends in PAL" and were calculated, considering the baseline PAL as the initial score. The sample was classified into four groups, as follows: G1 - Decreased PAL (insufficiently active → physically inactive; active → insufficiently; active → physically inactive); G2 - Increased PAL (physically inactive → insufficiently; physically inactive → active; insufficiently → active); G3 - Remained active (active → active; insufficiently → insufficiently); and G4 - Remained physically inactive (physically inactive → physically inactive). For statistical analysis, these four groups were categorized into two dichotomous variables (qualitative): (0) "Favorable behavior trend in PAL" and (1) "Not favorable behavior trend in PAL". A favorable behavior included groups 2 and 3 and a not favorable behavior included groups 1 and 4.

#### Socio-demographic and health variables

Independent variables selected from the Multidimensional Functional Assessment Questionnaire (BOMFAQ) for this study included gender, age group (65-69, 70-79, 80+ yrs), educational level (no formal, primary/secondary and college/university), body mass index, activities of daily living - ADLs (functional capacity), Mini-Mental (cognition) and dysthymia scores, falls and fractures [23]. All variables were relative to the beginning of the study.

Body weight (kg) and body height (cm) were used to calculate body mass index (kg/m^2^) [21]. A score between 24.9 and 18.5 kg/m^2 ^was considered as normal, overweight ranged from 25 and 29.9 kg/m^2 ^and obesity was defined as a BMI equal or above 30.00 kg/m^2 ^[29].

Functional capacity was measured using an ADL (activities of daily living) scale that consists of a checklist of 15 activities of daily living. Participants indicated whether they required assistance for the following: shopping, getting public transportation, caring for finances, taking medicines, walking a short distance, remaining continent, dressing, going to the toilet, grooming, cutting toe nails, bathing, eating, and getting in and out of bed. Each positive answer for requiring assistance to perform an activity is equivalent to one point. Those who reported the ability to manage all activities without help are classified as independent. A score from 1 to 3 indicates mild functional limitation, moderate ranges from 4 to 6 and severe is equal to or greater than 7 activities [4]. Therefore, high scores reflect a lower functional level.

Cognitive state was assessed by the Mini-Mental State Examination (MMSE), a brief 30-point screening test that is widely used to indicate cognitive impairment [30]. The MMSE test includes simple questions and problems in a number of areas: the time and place of the test, repeating lists of words, arithmetic, language use and comprehension, and basic motor skills. Scores range from 0 to 30. Higher scores on the MMSE indicate better cognitive performance. The cut-off point for detecting cognitive impairment in the illiterate elderly is 18 out of 19; in those who are educated, the best cut-off point is 24/25 [31].

Dysthymia was assessed using a score, validated for use among older residents in São Paulo. This 15-item schedule has forced-choice items with a "yes-no" format. The cut-off of five (5) positive answers classifies the possible presence of psychiatric disturbance, with higher scores reflecting a higher level of psychiatric disturbance [4,22].

In addition, all subjects were questioned on their medical history in regards to the occurrence of any fractures and falls in the last year.

#### Statistical analysis

Descriptive statistics (frequencies and percentages) were used to describe baseline characteristics of the sample and PAL after two years. Bivariate statistics and percentages were calculated first for the primary study variables. Variables thought to be related to PAL behavior trends over two years were examined for unadjusted association with the Pearson Chi-Squared test (bivariate statistics). All variables for which the association had *P < 0.05 *were retained for future analysis. A multivariate logistic regression model with Stepwise Forward methodology, considering *P < 0.10 *to enter and *P < 0.05 *to be removed, was then used to test the association between age, gender, educational level, body mass index, ADL and Mini-Mental State Examination scores, Dysthymia Screening and falls and fractures (independent variables) with behavior trends in PAL (dependent) after two years. Odds ratios (Ors) with a 95% confidence interval were reported. Analyses were conducted using SPSS software version 17.0.

## Results

### Sample characteristics

Baseline characteristics of respondents (n = 860) are shown in Table 1. There were significantly more women (69.2%) than men (30.8%). Furthermore, 49.6% were in the middle age group (70-79 years old), and approximately one-quarter of the cohort (20.9%) was included in the very old group (80+) (Table 1). The majority had completed primary school (49.1%). Nearly half of the elderly (44.7%) was classified as overweight (25-29.9 kg/m^2^). Overall, approximately 73% had a Mini-Mental score of 24+, 35.1% had no reported functional limitations and the majority showed lower prevalence of dysthymia (69.3%). In addition, 87.2% (n = 750) were classified as physically inactive, 4.2% (n = 36) as insufficiently active and 8.6% (n = 74) as physically active (Table 1).

**Table 1:** Distribution of baseline characteristics (n = 860) among older individuals in São Paulo, Brazil

Variables	n	%	Total
**Gender**			
Female	595	69.2	860
Male	265	30.8	
**Education**			
College/University	234	27.2	860
Primary/Secondary	422	49.1	
No formal	204	23.7	
**Age group (years)**			
65-69	229	29.5	776
70-79	385	49.6	
80+	162	20.9	
**PAL**			
Physically inactive	750	87.2	860
Insufficiently Active	36	4.2	
Active	74	8.6	
**BMI (kg/m**^**2**^**)**			
<24	136	31.5	432
25-29	193	44.7	
30+	103	23.8	
**ADL dependence score**			
0 (independent)	297	35.1	847
1-3 (mild)	291	34.4	
4-6 (moderate)	136	16.1	
7+ (severe)	123	14.5	
**MMSE**			
<24	628	73.0	860
24 +	232	27.0	
**Dysthymia Screening**			
Positive - 5+	235	30.7	765
Negative - <4	530	69.3	
**Falls (last year)**			
No	805	93.8	858
Yes	53	6.2	
**Falls**			
Yes no impact	225	26.2	858
Yes with impact	53	6.2	
No	580	67.6	
**Fractures (last year)**			
No	812	94.5	859
Yes	47	5.5	

PAL = physical activity level; ADL = activities of daily living; MMSE = Mini-Mental State Examination

### PAL at baseline and after two years

**Figure 1:**
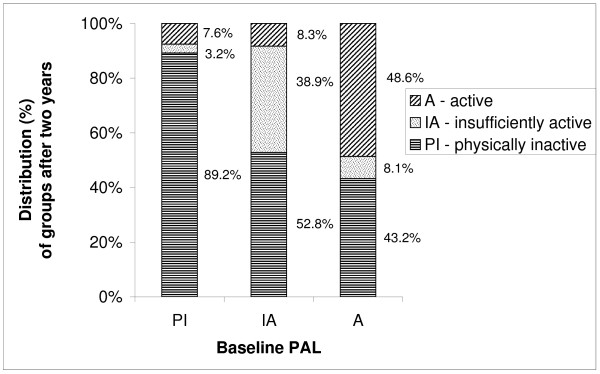
Distribution (%) of physical activity level after two years among older individuals in São Paulo, Brazil

Figure 1 shows the classification and distributions by percentage (%) of each group according to the PAL at baseline and after two years. According to these classifications, 89.2% of the individuals classified as physically inactive at baseline remained physically inactive, whereas only 7.6% became active after two years. Overall, most of the elderly who reported being insufficiently active at baseline became physically inactive (52.8%) and a small percentage (8.3%) from this group at baseline became active after two years. The majority of the active group remained active (48.6%) and only 8.1% became insufficiently active after two years (Figure 1).

### Behavior trends in PAL after two years

According to changes or maintenance in PAL of each group (physically inactive, insufficiently active and active) after two years, most of the elderly (84.4%) demonstrated a not favorable behavior trend in PAL, which included the participants who were and remained physically inactive or decreased PAL (insufficiently active → physically inactive; regularly active → insufficiently active). Moreover, only 15.6% presented a favorable behavior trend, which included the elderly who remained active or insufficiently active and those that increased their PAL (physically inactive → insufficiently active and insufficiently active → regularly active) (Table 2).

**Table 2:** Behavior trends in physical activity level after two years among older individuals in São Paulo, Brazil

Behavior trends in PAL	Frequency	Percent (%)
**Favorable**	134	15.6
**Not favorable**	726	84.4
**Total**	860	100.0

PAL = physical activity level

Favorable: increase or maintenance in activity level. Not favorable: decrease activity level or maintenance of physically inactive lifestyle.

### Health-related factors for a not favorable behavior trend in PAL (bivariate analysis)

In the bivariate analysis, using the Pearson Chi-Square test, gender, age, education level, ADL dependence and Mini-Mental scores, dysthymia scores and fractures and falls were all correlated significantly with behavior trends in PAL after two years (Table 3). Women were more likely to decrease PAL (88.1%; OR = 2.30; 95% CI = 1.58-3.35; *P *< 0.001) than men (76.2%). According to age, the oldest group (80+ years old) was more likely to decrease PAL (95.1%) vs. those who were 70 to 79 years (86.2%; OR = 2.27; 95% CI = 1.51-3.43; *P *< 0.001) and 65 to 69 years old (73.4%; OR = 6.99; 95% CI = 3.24-15.08; *P *< 0.001). Of the subjects with no formal education, 90.7% decreased in PAL over 2 years, almost 1.6 times the chance observed in those with a primary or secondary education (84.8%; OR = 1.56; 95% CI = 1.03-2.35; *P < 0.033*) and nearly three times the chance observed in those with higher education (superior degree/college/university) (78.2%; OR = 2.71; CI = 1.54-4.77; *P < 0.001*) (Table 3). BMI did not show a significant impact on trends in PAL over two years (data not shown). As expected, elderly who required some help for at least 4 of the 13 ADLs (ADL score) at baseline had 4 times more chance of displaying decreased PAL (90.4%; OR = 4.00; 95% CI = 2.27-7.01; *P < 0.001*) than those who did not report functional impairment (78.1%). Therefore, the more activities for which a subject required help, the less chance he/she had to have a favorable behavior trend in PAL (4-7 ADLs; 6.6%; OR = 4.00, 95% CI = 2.27-7.01, *P < 0.001*).

**Table 3:** Associations between health-related factors and not favorable behavior trend in PAL among older individuals in São Paulo, Brazil

Variable	Crude OR (95% CI)	*p*	Adjusted OR (95% CI)	*p*
**Gender**				
Male	1.00		1.00	
Female	2.30 (1.58-3.35)	0.000	2.50 (1.60-3.90)	0.000
**Age group**				
80+	1.00		1.00	
70-79	2.27 (1.51-3.43)		2.03 (1.29-3.18)	0.002
65-69	6.99 (3.24-15.08)	0.000	6.29 (2.70-14.68)	0.000
**ADL dependence score**				
0 (independent)	1.00		1.00	
1-3 (mild)	1.32 (0.88-1.98)	0.185	0.98 (0.60-1.59)	0.929
4-7+ (moderate/severe)	4.00 (2.27-7.01)	0.001	2.25 (1.20-4.21)	0.011
**Falls (last year)**				
No + Yes with no impact	1.00		1.00	
Yes with impact	3.24 (0.99-10.54)	0.051	6.89 (0.91-52.00)	0.062
**Education**				
No formal	1.00			
Primary/Secondary	1.56 (1.03-2.35)	0.001		
College/University	2.71 (1.54-4.77)	0.033		
**MMSE**				
24 + (24/25) (18-19)	1.00			
<24	2.07 (1.28-3.36)	0.003		
**Dysthymia Screening**				
Positive - 5+	1.00			
Negative - <4	1.53 (0.98-2.40)	0.062		
**Fractures**				
No	1.00			
Yes	4.37 (1.05-18.23)	0.043		

ADL = activities of daily living; MMSE = Mini-Mental State Examination

OR = odds ratio adjusted for gender, age and education; CI = confidence interval

Among the elderly group with an initial MMSE score less than 24, which is indicative of probable cognitive deficit, the proportion of individuals with favorable behavior trend in PAL was 9.5%, which is less than that among those indicating no cognitive deficit (17.8%; OR = 2.07; 95% CI = 1.28-3.36; *P < 0.003*) (Table 3).

The dysthymia scores was slightly associated with PAL behavior trends (*P < 0.061*). The proportion of individuals having a not favorable behavior trend in PAL in the elderly group that scored 5+ on the scale, which is not considered as probable dysthymia, was 17.7%, compared with 12.3% in the group with lower scores (OR = 1.53; 95% CI = 0.98-2.40; *P < 0.062*). A history of falls showed an association with PAL behavior trends. The percentage of elderly who experienced falls during the last year and had a not favorable behavior trend in PAL (94.3%; OR = 3.24; 95% CI = 1.00-10.54; *P < 0.051*) was 3 times higher than that of those who did not experience falls (83.3%) (Table 3).

Finally, the elderly who experienced fractures in their lifetime had approximately four times the chance of having a not favorable behavior trend in PAL (95.7%; OR = 4.37; 95% CI = 1.05-18.23; *P < 0.043*) compared with the elderly who did not experience fractures (83.7%) (Table 3).

Variables with a significant association in the bivariate analysis were first tested in a preliminary model. Educational level, Mini-Mental and dysthymia scores and fractures were excluded because they did not reach significance (data not shown). Age, gender, ADL scores and falls showed a significant association with the dependent variable of PAL behavior trends. Therefore, they were included in a progressively forward stepwise multivariate logistic regression model, which provided adjusted odds ratios (ORs) of PAL behavior trends over two years using a significance level of *P < 0.005 *and 95% confidence intervals (CIs). In this model, step 1 included age; step 2 included gender; step 3 included ADL scores; and finally, step 4 added falls.

### Health-related factors for a not favorable behavior trend in PAL (multivariate logistic regression model)

Results from the final model (Table 3) demonstrated that the risk of a not favorable behavior trend in PAL, which included the group who remained physically inactive and the group that displayed decreased PAL, in this cohort of older individuals was significantly increased if the individual was female (OR = 2.50; 95% CI = 1.60-3.89; *P < 0.000*), older (80 y vs. 65 y, OR = 6.29, 95% CI = 2.69-14.67; *P < 0.001 *and 80 y vs. 70-79 y OR = 2.03, 95% CI = 1.29-3.18; *P < 0.002*), dependent on help from others for activities in the ADL scores (moderate-severe = 4-7+ vs. 0 ADLs) (OR = 2.25, 95% CI = 1.20-4.21; *P < 0.011*) and experienced a history of falls with consequences (OR = 6.88, 95% CI = 0.91-52.01; *P < 0.062*) (Table 3). A history of falls with consequences was the most important explanatory variable for the risk of a not favorable behavior trend in PAL.

## Discussion

Over the last decade, interest in the impact of PAL on health among the elderly population has increased. Physical inactivity is common among elderly and this finding is particularly alarming considering that this population is greatly affected by noncommunicable diseases (NCDs). The current study found significant associations between behavior trends in physical activity level and age, gender, ADL scores and falls in a Brazilian urban community elderly population living in São Paulo state.

The prevalence of physical inactivity (87%) found in the baseline data was higher than that in another city in the south of Brazil (50.1%) [8] and in national data (56.5%) [9]. In addition, the prevalence of physical inactivity in leisure time among older adults was comparable to that of a large city in Vietnam (91%), but higher than that in four Lebanese districts (30%) [14,15]. In the U.S more than 30% of the adults above 70 years old were considered inactive in 2004 [32]. A possible explanation for this difference may be varying definitions of PA. The current study evaluated total PA considering only activities in leisure time. Some epidemiological studies in Brazil have demonstrated the importance of domestic and routines activities as part of the total PAL in the elderly population [7,8].

After two years, a decline in physical inactivity was observed in the elderly. However, when the data were evaluated more accurately, most of the elderly (84.4%) experienced a not favorable behavior trend in PAL, which included the participants who remained physically inactive and those who displayed decreased PAL (Insufficiently Active to Physically Inactive and Regularly Active to Insufficiently Active). In addition, only 15.6% (n = 134) remained active/insufficiently active or increased their PAL (Physically inactive → Insufficiently Active; Insufficiently Active → Regularly Active). Our finding is in line with other studies [32]. A study conducted in the U.S. showed a decline in physical inactivity (from 30% in 1994 to 24% in 2004) mainly in the age groups of 50 to 59 and 60 to 69 years old [32]. These findings suggest that despite the recognized benefits of PA for physical and psychological health and mortality [10-12,33], many older adults remain physically inactive and maintain this negative behavior. Additionally, considering that initiating activities in leisure time even in midlife increases the probability of successful survival or exceptional overall health in later life [33], public health efforts should emphasize the promotion of physical activity and identify the factors that limit physical activities among older adults.

Considering age, the present data are in agreement with information presented in the literature [7,8,14-17,19,34], which shows a gradual decrease in physical activity participation with an increase in age. In Poland, for example, the risk of leisure time physical inactivity was significantly higher among persons older than 64 years of age. Our study also demonstrated that the oldest elderly have a lesser chance of becoming physically active or insufficiently active despite their initial PAL, which may be attributable to a high proportion of the elderly requiring assistance in activities of daily living and to a decrease in work-related activities [34].

In the present study, men were more active than women and improved their PAL after two years. Similar findings have been extensively reported [7,16,20,34,35]; however, most of them, including our research, did not evaluate household activities, which contribute considerably to the overall physical activity level among elderly women. A study conducted in Vietnam observed that elderly women were more active than men [15]. This difference could be explained by the fact that a high proportion of women are involved in household activities even in later life, contributing to the overall PAL [15].

There is strong evidence supporting an association between educational level and physical activity in the elderly [7,14-16,36,37]. Our study demonstrated that elderly individuals with a poor education were more physically inactive in leisure time. Furthermore, after 2 years, they became physically inactive or insufficiently inactive despite their initial physical activity level. There are several possible explanations for this result. First, those with a lower level of education might have a lack of knowledge of PA benefits and fewer opportunities to participate in physical activities during leisure time. Second, in developing countries, occupation/work activities are important components of total activity level in addition to leisure-time activities, even among the elderly [14,16].

The relationship between functional capacity and PAL has been demonstrated in several studies [19,38-40]. Data from "The Women's Health and Aging Study" have demonstrated that physical inactivity is more frequent among women with a lower functional capacity [39]. A similar result is seen in the south of Brazil. Moderate to severe difficulties in performing activities of daily living were associated to physical inactivity among elderly women [19]. These results are in agreement with the results from the current study, which indicates that more than four limitations in ADL score is positively associated with less physical activity. Furthermore, after two years, the prevalence rates of becoming physically inactive increased with rising difficulties in ADL. Therefore, there are strong evidences showing the relationship of exercise with functional capacity [19,38-40].

A strong association was found between physical inactivity and the Mini-Mental State Examination in the first analysis. The majority of subjects that experienced a decrease in PAL after two years presented lower values for cognitive status. However, when controlled by other variables, this aspect did not show a correlation with PAL. These results are contrary to findings from other countries in which low levels of physical activity were associated with poorer cognitive performance [41,42]. One of the analyzes of the Duke Longitudinal Study, Texas, U.S., showed lower scores in cognitive tests in the elderly group that retired and remained sedentary compared with the group that retired but continued performing regular physical activities. Furthermore, those that retired and later adopted a sedentary lifestyle presented a greater risk of brain-vascular disease and possible cognitive decline compared to the group that retired but continued performing regular physical activities [41]. Among 18,766 elderly nurses between 70 and 81 years old in the United States, long-term regular physical activity, including walking, was associated with significantly better cognitive function and less cognitive decline [43]. However, the inverse relationship has not been well established in prospective studies [44].

No strong associations were found between physical inactivity and BMI or the dysthymia screen. Likewise, no significant relationship was reported between these variables and behavior trends in PAL over two years.

Some cross-sectional studies found significant association between PAL and BMI [14,17]; nevertheless, the present results are in agreement with other Brazilian studies [8,45]. Despite the benefits obtained by exercise (an increase in rest metabolic rate and a decrease in risk of cardiovascular diseases), there are no data that show a change in adiposity or in body weight of the elderly with an exercise program that does not include dietary restriction [46]. This result may explain the findings of our study, including the fact that we did not find changes in the BMI. However, we cannot prove this hypothesis because in our study, dietary habits and other variables that may influence body composition were not considered longitudinally.

A positive relationship between physical inactivity and psychological well-being in the elderly without a clinical disorder has been reported in meta-analysis studies [47]. However, data from other studies have demonstrated conflicting results [47-49]. A recent study showed that the elderly with emerging depression in Amsterdam, the Netherlands, tended to change more often to a sedentary lifestyle than those without depression, although no causal relationship was demonstrated [49]. Because depression is closely related to functional decline in older adults [50], when ADL were controlled in the final logistic regression model, no strong association was found between scores of dysthymia screen and behavior trends in PAL. Additionally, response bias and cohort differences are problems related to self-report, and perceptions of well-being differ in older cohorts [51]. Furthermore, most of the findings are from studies that have evaluated the influence and benefits of an active lifestyle for depression and among younger groups.

Our finding of correlation between falls and a not favorable behavior trends in PAL is in line with other studies [52-54]. In a cross-sectional study Zijlstra and colleagues [53] verified that multiple falls were independently associated (OR = 4.64; 95% CI = 3.73-5.76) with the avoidance of activity among 4,031 community-living elderly. A study conducted in Belgium demonstrated that falls were correlated with the avoidance of activities in community-living elderly between 61 and 92 years old, both in cross-sectional and in longitudinal analyses [54]. An interesting hypothesis for these findings may be the gradual development of avoidance behavior at the mobility level, which results in a deterioration of physical abilities and falls at home in the long term. Previous cross-sectional studies have shown a correlation between falls and fear of falling, but it is unclear which occurs first. Several studies [53,54] have pointed out that fear-related avoidance of activities may have negative effects on physical abilities and may also be predictive of future falls. Falls are a common and important problem in older community-dwelling adults. One of the major consequences of falls is avoidance activities, which consequently decrease PAL. Future research should focus on strategies to prevent falls within this population.

Although we did not observe an independent effect of fractures on PAL behavior trends, some studies have shown the importance of fractures in decreasing PAL [55,56]. Despite the potential benefits associated with exercise after a fracture, elderly from *Baltimore Hip Studies *who sustained hip fractures were not likely to engage in regular exercise (resistive or aerobic). Furthermore, many factors may influence older adults' motivation and willingness to exercise, such as poor health, disability, lack of knowledge about exercise and its benefits, lower sense of self-efficacy for exercise [56], and fear of falling and injury [53,54].

Noncommunicable diseases were not analyzed in the present study but may be associated with and/or explain changes in some variables after a follow-up period.

Physical activity involvement, mainly the increase in physical activity level and the maintenance of an active/insufficient level, must be emphasized as a health-promotion intervention policy for this group.

The limitations of our study should be considered when interpreting the results. First, the lack of a question regarding the intensity of physical activities must be considered. However, for the most part, studies have shown that elderly people tend to adopt physical activities of moderate intensity. Additionally, we did not consider or measure the influence of other variables (confounders) that may be related to body mass index, functional capacity, cognitive and dysthymia screen, falls and fractures (statistical analysis). Another limit was the short follow-up period (two years). Furthermore, we did not analyze other components of overall physical activity level. Individuals who are inactive during leisure time may be more active in other contexts. In developing countries, household and routine activities contribute greatly to total PA. In addition, because other studies used different definitions of physical inactivity, instruments and cut-off points, comparisons are difficult. Finally, the difference of almost twenty years between the first stage of the Project and the publication of the results is not considerable, while our main purpose was to verify the relationship between health-related factors and PAL trends.

## Conclusions

This study demonstrated that gender, age, dependence in ADL and falls (after adjustment for some potential confounders) were associated with PAL and its trends over two years. Being older and female with poor functional capacity and having experienced falls contributes to a not favorable behavior trend in PAL among the elderly longitudinally. Our findings highlight the need for health-promotion programs that target these factors related to PAL, reducing barriers to achieve desired changes. The development and implementation of programs to promote a physically active lifestyle must be encouraged, even in later phases of life.

## Competing interests

The authors declare that they have no competing interests. The organizations funding this study had no role in the design or conduct of the study; in the collection, management, analysis, or interpretation of the data; or in the preparation, review, or approval of the manuscript.

## Authors' contributions

LRR had full access to all of the data in the study and takes responsibility for the integrity of the data and the accuracy of the data analysis. He was involved with study concept and design, acquisition of data, analysis and interpretation of data, drafting of the manuscript, and critical revision of the manuscript for important intellectual content. SMMM was involved with analysis and interpretation of data, drafting of the manuscript, and for important intellectual content. MCSAR provided data analysis advice and critical revision of the manuscript. MTF was involved with acquisition of data, statistical analyses and interpretation of data, drafting of the manuscript, and critical revision of the manuscript. All authors read and approved the final manuscript.

## Pre-publication history

The pre-publication history for this paper can be accessed here:

http://www.biomedcentral.com/1471-2458/10/690/prepub
